# Towards the optical cochlear implant: optogenetic approaches for hearing restoration

**DOI:** 10.15252/emmm.201911618

**Published:** 2020-03-30

**Authors:** Alexander Dieter, Daniel Keppeler, Tobias Moser

**Affiliations:** ^1^ Institute for Auditory Neuroscience and InnerEarLab University Medical Center Göttingen Göttingen Germany; ^2^ Göttingen Graduate School for Neurosciences Biophysics and Molecular Biosciences University of Göttingen Göttingen Germany; ^3^ Auditory Neuroscience and Optogenetics Laboratory German Primate Center Göttingen Germany; ^4^ Auditory Neuroscience Group Max Planck Institute of Experimental Medicine Göttingen Germany; ^5^ Cluster of Excellence “Multiscale Bioimaging: from Molecular Machines to Networks of Excitable Cells” (MBExC) University of Göttingen Göttingen Germany

**Keywords:** cochlear implant, gene therapy, hearing restoration, optogenetics, Synthetic Biology & Biotechnology, Genetics, Gene Therapy & Genetic Disease, Neuroscience

## Abstract

Cochlear implants (CIs) are considered the most successful neuroprosthesis as they enable speech comprehension in the majority of half a million CI users suffering from sensorineural hearing loss. By electrically stimulating the auditory nerve, CIs constitute an interface re‐connecting the brain and the auditory scene, providing the patient with information regarding the latter. However, since electric current is hard to focus in conductive environments such as the cochlea, the precision of electrical sound encoding—and thus quality of artificial hearing—is limited. Recently, optogenetic stimulation of the cochlea has been suggested as an alternative approach for hearing restoration. Cochlear optogenetics promises increased spectral selectivity of artificial sound encoding, hence improved hearing, as light can conveniently be confined in space to activate the auditory nerve within smaller tonotopic ranges. In this review, we discuss the latest experimental and technological developments of cochlear optogenetics and outline the remaining challenges on the way to clinical translation.

## Graphical abstract

In this review, A. Dieter, D. Keppeler and T. Moser summarize the state of the art and progresses made in optical cochlear implantation using ontogenetic technology and discuss the challenges for translation into patients with hearing loss.

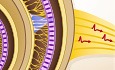

**Glossary**
Adeno‐associated virus (AAV): Single‐strand DNA virus, considered to be non‐disease‐causing, often used as vector of choice for expressing transgenes of interest for gene‐therapeutic approaches. AAVs are engineered to not integrate their DNA in the host genome.
Auditory Brainstem Response (ABR): Evoked population response reflecting summed synchronized action potentials in the auditory nerve, various auditory brainstem nuclei, and the auditory midbrain.
Channelrhodopsins: Light‐gated ion channels originally found in green algae. When introduced into excitable cells (such as neurons), channelrhodopsins enable precisely controlled light‐induced action potential generation.
Cochlear implant (CI): Neuroprosthetic device which directly stimulates the auditory nerve and thereby partially restores hearing in patients suffering from profound sensorineural hearing loss.
Cochlear optogenetics (in this review): Optogenetic stimulation of spiral ganglion neurons.
Cochlea: Spiral‐shaped hearing end organ of the inner ear.
Dynamic range: Range of stimulus intensities which can be encoded.
Immune‐privileged: Sites within the body in which it is considered that foreign molecules are not recognized and neutralized by the immune system.
Inferior Colliculus (ICC): Structure of the midbrain, whose central nucleus (ICC) is characterized by its prominent tonotopic organization.
Light‐emitting diode (LED): Semiconductor light source which emits photons when electric current is applied.
Mechanotransduction: Conversion of mechanical stimulation such as pressure waves into electric signals by mechanosensory hair cells.
Modiolus: Central axis of the cochlea housing the spiral ganglion.
Monte Carlo ray tracing: Stochastic modeling of the beam path of a large number of photons.
Optoelectronic: Electronic devices emitting or detecting light.
Optogenetics: Genetic modification of biological tissue enabling control of cells by light.
Organ of Corti: Sensory organ of the inner ear, housing inner and outer hair cells as well as various supporting cells.
Ossicles: Three bones (malleus, incus, and stapes) in the middle ear which amplify and relay pressure waves from the outer ear arriving at the eardrum to the inner ear via the oval window.
Otocyst: Embryonic progenitor of the inner ear which later on will differentiate into the cochlea and the vestibular system.
Photocurrents: Ionic currents mediated by light‐gated ion channels upon illumination.
Phototoxicity: Damage of cells or tissue evoked by intense exposure to light.
Ribbon synapses: Specialized synapses in the inner ear and retina which are characterized by electron‐dense structures (ribbons) which tether synaptic vesicles to presynaptic active zones.
Rosenthal's canal: Cavity in the modiolus housing the cell bodies of spiral ganglion neurons.
Scala tympani: Perilymph‐filled intracochlear cavity extending from the round window to the helicotrema.
Sensorineural hearing loss: Hearing loss resulting from dysfunction of the cochlea and/or spiral ganglion.
Spectral selectivity: Precision by which acoustic, electrical, or optogenetic stimulation, the cochlea can encode sound frequency in the auditory system.
Spiral ganglion neurons (SGNs): Bipolar neurons housed in Rosenthal's canal in the modiolus who innervate hair cells and whose axons form the auditory nerve, projecting to the cochlear nucleus in the auditory brainstem.
Tonotopy: Place‐frequency code in the auditory system.
Trafficking signals: Sequence of amino acids derived from inward rectifying potassium channels which support protein export from the endoplasmic reticulum (ES) and trafficking to the plasma membrane (TS).
Transduction: Gene transfer by viral vectors.
Transuterine injection: Method to introduce virus suspension for gene therapy into the host organism during the embryonic stage.
Vector strength: Measure of periodicity of a neuronal response to an outside periodic signal.
Waveguides: Conductors which guide electromagnetic waves to their target structures, also referred to as optical fibers.

## A primer to acoustic, electric, and optogenetic hearing

### Synaptic sound encoding in the cochlea

**Figure 1:**
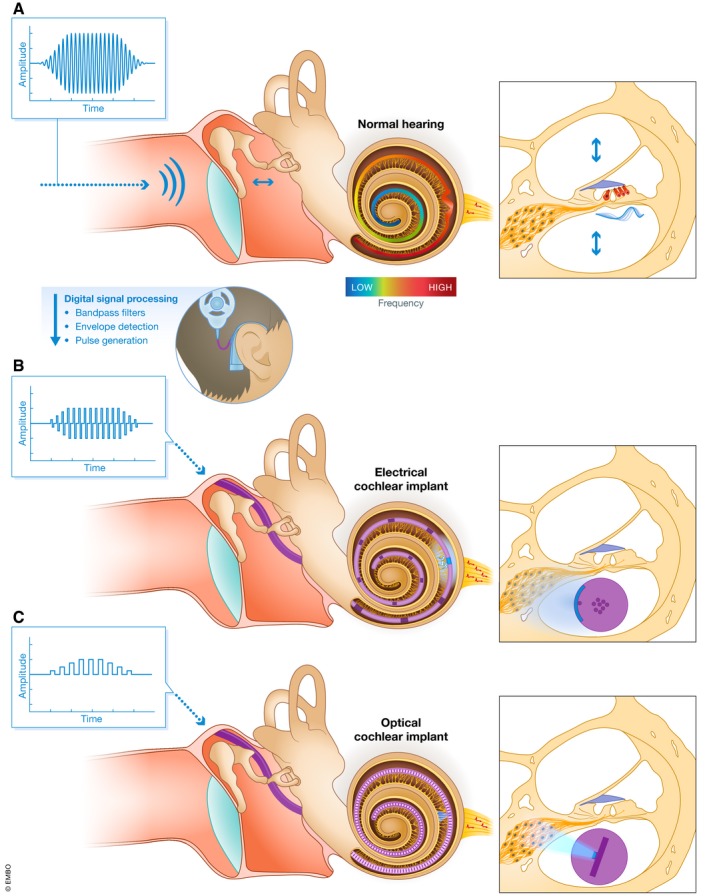
Natural vs. artificial sound encoding in the cochlea (A) Sound pressure waves in the air (left) travel along the ear canal and are relayed via the ossicles into the intracochlear fluid, where they are decomposed in a frequency‐dependent manner (center). A so‐called traveling wave along the basilar membrane activates mechanosensitive hair cells (red) in the organ of Corti at the respective cochlear location and thereby starts the information flow in the auditory system via synaptic transmission to SGNs (yellow, right). The precise frequency‐place mapping (tonotopy) is visualized through the color‐coded basilar membrane (see color bar). (B, C) Acoustic signals are analyzed by an external processor, which extracts predominant frequencies and corresponding amplitudes of the signal. While frequencies are mapped to distinct stimulation sites (electrodes in the eCI or light emitters in the future oCI) dependent on their tonotopic position, the signal amplitude determines stimulation intensity. SGNs around the tonotopic region that would be activated by hair cells for a given sound frequency in physiological hearing (A) are then directly activated with electric current (B) or light (C). Since light can be better confined in space than electric current, oCIs promise to activate the spiral ganglion with higher spatial precision (B vs. C, right).

Acoustic signals, including human speech, are composed of various air pressure waves, and thus defined by physical features such as frequency and amplitude that fluctuate in time. The cochlea of the inner ear functions as a spectral analyzer of these features: Due to its intrinsic mechanical properties, different frequency components are decomposed along the cochlea and the cochlear traveling waves—waves in the intracochlear fluids which have been relayed from air pressure waves via the ossicles—activate inner and outer hair cells (IHCs and OHCs) at different cochlear locations, establishing a frequency map in the cochlea (also known as tonotopic axis). The amplitudes of these frequency components determine both the extent of hair cell activation at the respective cochlear location and the spread of hair cell activation along the tonotopic axis (Fig 1A; von Békésy & Wever, 1960; Chatterjee & Zwislocki, 1998). OHCs amplify and sharpen the traveling waves for soft sounds (Ashmore, 2008). IHCs employ sophisticated ribbon synapses (Moser *et al*, 2019) to transmit the sound information to the encoding spiral ganglion neurons (SGNs), the primary afferent neurons of the auditory system. Spike rate and the number and identity of spiking SGNs are thought to encode sound amplitude. The cochlear location of SGN activation encodes the sound frequency, making use of the intrinsic place‐frequency code of the cochlea. Finally, spike timing transmits information on the temporal structure of a sound and—for low frequencies—on its frequency. The tonotopic organization is kept throughout the auditory pathway up to the cortex.

### Electrical hearing—a success story of neuroprosthetics yet with limitations

Hearing impairment is the most prevalent sensory deficit and has major socioeconomic impact. According to the World Health Organization (WHO), 466 million people suffer from disabling hearing loss (HL), mainly resulting from cochlear disorders such as genetic, noise‐induced, or age‐related hearing loss. HL often causes social isolation and results in a yearly economic impact of 750 billion US$ spent on prevention, identification, and treatment of HL (WHO, 2019). Therapies are currently being investigated and include regenerative approaches such as pharmacologically induced trans‐differentiation of cochlear supporting cells into hair cells (Samarajeewa *et al*, 2019) as well as gene (Ahmed *et al*, 2017) and stem cell therapies (Roccio *et al*, 2019). However, these approaches are still far from clinical translation and not suitable for all forms of HL. Thus, the current state‐of‐the‐art means of rehabilitation for HL are hearing aids and electric cochlear implants (eCI). The eCI is provided in case of profound hearing loss or deafness. It consists of an external component—consisting of a microphone and a processor—as well as an internal component, containing a linear electrode array placed along the cochlear axis that emanates from the stimulator (Fig 1B). The sound processor extracts predominant frequency components from the surrounding auditory environment and maps them to the eCI electrodes located at different positions along the tonotopic axis. By electrically stimulating SGNs around these electrodes, eCIs utilize the intrinsic place‐frequency code of the cochlea and patients perceive a pitch that roughly matches the frequencies which are physiologically coded at these cochlear positions. Electrical sound encoding restores open speech comprehension in most users. However, massive current spread from each of the active electrodes recruits rather large populations of SGNs, which limits the precision by which eCIs can utilize the place‐frequency code of the cochlea (Shannon, 1983; Kral *et al*, 1998). Furthermore, the output dynamic range of eCIs is low, which limits intensity coding of electrical hearing (Zeng, 2004; Miller *et al*, 2006). These intrinsic limitations of electrical sound encoding ultimately limit hearing restoration and, importantly, make it difficult for eCI users to understand speech in background noise (Caldwell *et al*, 2017).

### Optical hearing—a promising alternative for improved hearing restoration

Using light as an alternative strategy for artificial sound encoding, it might be possible to overcome the limitations of eCIs. As light can be conveniently confined in space, it enables SGN stimulation with higher spatial selectivity, resulting in improved spectral selectivity (Fig 1C; Izzo *et al*, 2007; Richter *et al*, 2011; Hernandez *et al*, 2014; Jeschke & Moser, 2015; Moser, 2015). Optical stimulation of the cochlea got started by Richter and colleagues, who have used pulsed infrared lasers to stimulate SGNs (Izzo *et al*, 2007). However, these experiments revealed a high‐energy threshold for neural activation (starting at 15 μJ per pulse; Izzo *et al*, 2007; Tan *et al*, 2015) and the utility of direct infrared stimulation of SGNs has been challenged by studies in other laboratories (Teudt *et al*, 2011; Thompson *et al*, 2015; Kallweit *et al*, 2016; Baumhoff *et al*, 2019).

Lower light requirements as compared to infrared neural stimulation and a molecularly defined mechanism of neural activation by light are offered by optogenetics: One and a half decades ago, it has been demonstrated that light‐gated ion channels found in green algae, called Channelrhodopsins (ChRs; a subtype of microbial opsins), mediate light‐driven action potentials in mammalian neurons (Nagel *et al*, 2003; Boyden *et al*, 2005). Since then, the optogenetic toolbox has been extended tremendously and now allows for cell type‐specific neural control with high spatial and temporal precision by a tunable mechanism, which raises hope to restore neural function in disorders such as Parkinson's disease (Delbeke *et al*, 2017), epilepsy (Tønnesen & Kokaia, 2017), cochlear (Hernandez *et al*, 2014), and retinal degeneration (Scholl *et al*, 2016). Some of these tools, which might be interesting candidates for hearing restoration, are summarized in Table 1. By optogenetically rendering SGN light sensitive, a promising implementation of the optical cochlear implant (oCI) has become feasible. This requires efficient, stable, and safe means of expressing appropriate optogenetic tools in the SGNs. Currently, the local administration of non‐pathogenic adeno‐associated viruses (AAVs) to the cochlea as vectors for transducing SGNs emerges as the method of choice. Then, even broad neuronal promoters, such as the human synapsin promoter, can be employed to selectively express optogenetic tools in SGNs as they represent the only neuronal population with cell bodies localized in the cochlea (Wrobel *et al*, 2018). The rate of transduction is co‐determined by the efficacy of the route of administration, the type and number of AAV particles, the strength of the promoter, and the accessibility and susceptibility of the target cells for the viral vector. Note that viral transduction alone, i.e., without expression of the optogenetic tool, does not render SGN light sensitive and that future optogenetic hearing restoration requires the development of both the medical device oCI and the optogenetic manipulation (gene therapy). Here, we review the latest progress of biomedical and optoelectronic development of oCIs, and subsequently discuss challenges remaining on its way toward clinical translation.

**Table 1:** Candidate opsins for optogenetic hearing restoration

ChR variant	τ_off_ RT (ms)	τ_off_ BT (ms)	λ (nm)	References
ChR2	9.4–10	3.0	~470	Klapoetke *et al* (2014), Keppeler *et al* (2018), Mager *et al* (2018)
CatCh	16.3	–	474	Kleinlogel *et al* (2011)
Chronos	3.0–3.6	0.76	~490	Klapoetke *et al* (2014), Keppeler *et al* (2018)
CheTa	4.4	–	~500	Gunaydin *et al* (2010)
f‐Chrimson	5.7	3.2	594	Mager *et al* (2018)
Vf‐Chrimson	2.7	1.6	594	Mager *et al* (2018)

Summary of closing kinetics at room (RT) and body temperature (BT) and peak action spectrum for selected ChR variants. The temporal fidelity of optogenetic stimulation with a subset of these opsins in the auditory system is displayed in Fig 3.

## Hearing with light—biological proof of feasibility for cochlear optogenetics

Toward the development of optical cochlear implants, two important objectives need to be met: First, the general feasibility of cochlear optogenetics needs to be demonstrated, including optical activation of the auditory nerve and subsequent signal propagation along the auditory pathway, as well as stimulus perception by the animal. Ideally, longitudinal experiments covering the lifespan of the model organism should be performed and hearing should be restored in animal models of human sensorineural hearing loss. Second, improved performance of optical over electrical sound encoding must be demonstrated, since clinical translation of the oCI can only be justified if a major improvement in hearing restoration is to be expected. Recent experiments employing optical fiber‐based cochlear optogenetics in transgenic mice (Hernandez *et al*, 2014) as well as rodents whose SGNs have been transduced with adeno‐associated viruses (AAVs) made progress toward these goals (Hernandez *et al*, 2014; Duarte *et al*, 2018; Keppeler *et al*, 2018; Mager *et al*, 2018; Wrobel *et al*, 2018; Dieter *et al*, 2019).

### Optogenetic activation of the auditory system

In a first proof‐of‐principle study (Hernandez *et al*, 2014), optogenetic activation of the auditory system was reported in transgenic mice broadly expressing ChR2 in neural structures under the *Thy1.2* promoter (Arenkiel *et al*, 2007). The feasibility of optogenetic excitation of the auditory system was first demonstrated by recordings of auditory brainstem responses (ABR): ABRs are far‐field potentials, reflecting the synchronous activation of the auditory system up to the auditory midbrain and are typically characterized by five waves (originating from activation of the auditory nerve, a set of nuclei in the auditory brainstem and finally the inferior colliculus (Henry, 1979; Land *et al*, 2016)) when elicited by acoustic stimulation (aABRs). Using optogenetic (oABR) stimulation, it could be demonstrated that cochlear optogenetics is capable of evoking potentials of up to 2.5 mV amplitude. Such potentials by far exceeded the amplitudes of aABRs (~5 μV) and were closer to electrically evoked (eABR) amplitudes (Hernandez *et al*, 2014). Furthermore, optically evoked potentials of up to 600 μV amplitude could be evoked in mice upon transuterine injections of AAV2/6 carrying a calcium‐translocating ChR2 variant (CatCh; Kleinlogel *et al*, 2011) under the human synapsin promoter into the otocyst during embryonic days 11–12, which led to transduction of SGNs primarily in the high‐frequency base of the cochlea (Hernandez *et al*, 2014).

Follow‐up studies of mice whose spiral ganglion has been postnatally transduced by intracochlear virus injections corroborated optogenetic activation of the auditory pathway. Injections of AAV2/6 carrying the ChR variant f‐Chrimson under the human synapsin promoter enabled oABRs with amplitudes of up to ~10 μV, similar to aABRs (Mager *et al*, 2018). Likewise, injections of the potent AAV‐PHP.B (an engineered capsid which has been demonstrated to transduce neurons with ~40‐fold higher efficiency as, e.g., AAV9) carrying the fastest naturally occurring opsin Chronos, under the human synapsin promoter (Klapoetke *et al*, 2014) which was optimized by adding trafficking signals which promote the integration of light‐gated ion channels into the plasma membrane, enabled oABRs with amplitudes comparable to aABR (Keppeler *et al*, 2018). In both studies, high transduction rates (on average greater than 60%) across all tonotopic regions were observed in the injected ear. Importantly, no obvious SGN loss was found (Keppeler *et al*, 2018; Mager *et al*, 2018). In addition, a problem of the approach was noted: Substantial spread of virus was evident by ChR expression in the contralateral ear. In mice that had been transduced with a Chronos construct lacking the trafficking signals, opsin expression in the cell membrane was relatively weak and oABR amplitudes were smaller (Keppeler *et al*, 2018). This is in agreement with a parallel study using injections of the potent AAV‐Anc80L65 (an *in silico* designed, evolutionary intermediate capsid with high transduction efficiency) carrying Chronos under the *CAG* promoter, which reported oABRs with mean amplitudes of 0.65 μV upon blue light illumination of the auditory nerve (Duarte *et al*, 2018). In these studies, expressing channelrhodopsins via postnatal SGN transduction, latency (~1 ms), waveform (3–5 waves), and amplitude (<1 μV to 10 μV) of oABRs elicited by strong stimuli were much more comparable to those of mouse aABRs (approximately 1.4 ms, typically 4 waves, up to 8 μV; Duarte *et al*, 2018; Keppeler *et al*, 2018; Mager *et al*, 2018) than in the proof‐of‐principle study (Hernandez *et al*, 2014). This most likely reflects optogenetic auditory nerve activation with higher specificity when mediated by local virus injections into the postnatal cochlea as compared to broad transgenic ChRs in all neuronal structures.

In order to get closer to a translational approach, virus‐mediated optogenetic SGN manipulation was established in adult Mongolian gerbils (Wrobel *et al*, 2018), which serve as an important animal model for auditory research given they exhibit low‐frequency hearing more similar to humans. AAV2/6 carrying a gene encoding for CatCh under control of the human synapsin promoter was injected directly into the modiolus, the bony compartment housing the SGNs, of adult Mongolian gerbils. This yielded transduction of SGNs across all tonotopic regions with an average rate of 30% and some SGN loss (25%). The SGN loss was likely due to the intramodiolar pressure increase rather than neurotoxicity of the AAV suspension as it was similarly found upon saline injection. The achieved CatCh expression enabled oABRs in approximately half of the injected animals of up to ~1.3 μV (Wrobel *et al*, 2018). Even though oABR amplitudes were lower than the ones observed in postnatally transduced mice (which most likely can be attributed to less efficient SGN transduction and the thicker skull in adult gerbils; Keppeler *et al*, 2018; Mager *et al*, 2018), oABR amplitudes are comparable to aABR amplitudes evoked by acoustic clicks of 40 dB SPL in non‐injected gerbils (Wrobel *et al*, 2018).

Across all animal models and transduction methods, oABR amplitudes increased and latencies decreased with stronger illumination of the cochlea, suggesting recruitment of more spiral ganglion neurons with higher temporal precision when using higher light intensities (Duarte *et al*, 2018; Keppeler *et al*, 2018; Mager *et al*, 2018; Wrobel *et al*, 2018). Optogenetic activation of the rodent auditory pathway has further been confirmed by electrophysiological recordings of single putative SGNs in AAV‐injected and transgenic animals (Hernandez *et al*, 2014; Keppeler *et al*, 2018; Mager *et al*, 2018), by local field potentials and multi‐unit activity in the auditory midbrain (Hernandez *et al*, 2014; Dieter *et al*, 2019), as well as single‐neuron activity of primary auditory cortex (Wrobel *et al*, 2018).

### Spectral selectivity of cochlear optogenetics

The first evidence for improved spatial (and thus spectral) selectivity of optogenetic over electrical SGN stimulation was demonstrated by recordings of local field potentials in the central nucleus of the inferior colliculus (ICC) in the auditory midbrain of ChR2‐transgenic mice for suprathreshold optical, electric and acoustic stimulation of the auditory nerve (Hernandez *et al*, 2014). The ICC is characterized by a remarkably conserved tonotopy, and therefore, an assessment of neuronal activation in the ICC allows direct inference about the spread of excitation in the cochlea. Using current source density analysis, which reflects excitatory inputs of ICC neurons, activation in high‐frequency layers of the ICC was revealed upon illumination of the cochlear high‐frequency base by an optical fiber (Hernandez *et al*, 2014). Optogenetic stimulation was significantly more confined in space (1.74‐fold) than single‐channel monopolar electrical stimulation and was statistically indistinguishable from pure tone acoustic stimulation (31 kHz, 80 dB SPL; Hernandez *et al*, 2014).

**Figure 2:**
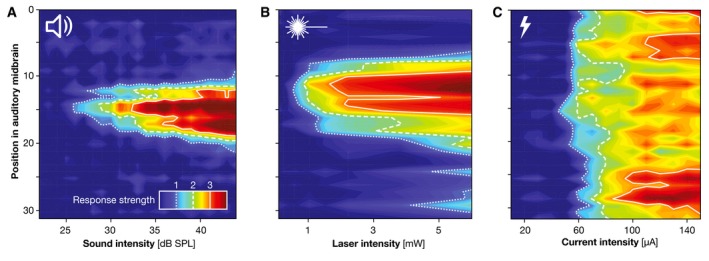
Spectral selectivity in the auditory system Neural activation (color‐coded in d’ units) across the auditory midbrain (ordinate) upon (A) acoustic, (B) optogenetic, and (C) electric activation of the auditory nerve with increasing stimulus intensity (abscissa). While acoustic stimulation and optogenetic stimulation of the cochlea activate the auditory midbrain with relatively high spatial selectivity (A, B), electric stimulation leads to broader activation (C). Figure is plotted using data published by Dieter *et al* (2019).

In a more recent study, spectral selectivity of natural and artificial SGN stimulation has been analyzed by multi‐channel recordings of neuronal clusters in the ICC of Mongolian gerbils (Dieter *et al*, 2019). Optogenetic stimulation was performed with up to three laser‐coupled optical fibers placed at distinct positions along the cochlear tonotopic axis of gerbils whose mature auditory nerve was virally transduced with CatCh. Upon optical stimulation of SGNs at low‐, medium‐, or high‐frequency positions in the cochlea, spatially selective neuronal activity has been observed in tonotopically corresponding regions of the ICC (Dieter *et al*, 2019). The activity pattern in the ICC critically depended on the precise projection of light onto SGNs. Acoustic stimulation using pure tones and electrical stimulation were done in parallel in naïve animals. Monopolar electrical stimulation and bipolar electrical stimulation were achieved using 4‐channel clinical‐style eCIs inserted via the round window. An activity‐based analysis at similar levels of ICC activation upon acoustic, optogenetic, and electric SGN stimulation enabled comparison of the spectral selectivity across stimulus modalities. It was found that optogenetic stimulation was spatially more selective than monopolar electrical stimulation at all activation strengths and outperformed bipolar electrical stimulation at medium and high activation strengths (as much as 2.04‐ and 1.94‐fold, respectively). Furthermore, optogenetic SGN stimulation was found to be as selective as acoustic stimulation at low and modest activation levels, but caused broader activation at higher stimulus intensities (Fig 2; Dieter *et al*, 2019).

Further evidence for high spectral selectivity was obtained by Monte Carlo ray‐tracing simulations: Here, the beam paths of millions of photons in the cochlea delivered via optical fibers (reconstructed by X‐ray tomography) based on optical properties of intracochlear tissues (derived from literature) under the conditions of the biological experiments were modeled (Wrobel *et al*, 2018; Dieter *et al*, 2019). Besides corroborating the claim for high spectral selectivity, the ray‐tracing model suggests that the selectivity of cochlear optogenetics can be even further improved by the implementation of light sources with smaller emitting surface, lower numerical aperture, and optimized position relative to the neural target tissue (Wrobel *et al*, 2018). Taken together, increased spectral selectivity of optogenetic over electric auditory nerve stimulation suggests that cochlear optogenetics can, indeed, increase the frequency resolution of artificial sound encoding.

### Dynamic range of optogenetic SGN stimulation

Besides poor transmission of spectral information, electrical sound encoding of stimulus intensity is limited, too. While the dynamic range of acoustic stimulation amounts up to 120 dB, the output dynamic range of eCI coding is restricted to 10–20 dB (Rubinstein, 2004; Zeng, 2004). This is not surprising as the large dynamic range of acoustic hearing is enabled by several cochlear mechanisms: OHC‐mediated amplification and compression of the traveling wave, diversity of synaptic sound encoding at each tonotopic position, and various sensory and neural adaptation mechanisms. These mechanisms are lacking when the eCI directly stimulates SGNs in the deaf cochlea. In this case, the dynamic range reflects that of electrically driven individual SGN firing (1–2 dB) and the recruitment of SGN populations at further distances from the electrode, which show similar current thresholds (Viemeister, 1988; Miller *et al*, 2006). For optogenetic SGN stimulation, dynamic ranges of 10–20 dB were reported, based on the growth function of oABRs, which increased in amplitude for stimulation intensities of more than one order of magnitude (Keppeler *et al*, 2018; Mager *et al*, 2018; Wrobel *et al*, 2018). In a recent study based on multi‐unit activity in the auditory midbrain, dynamic ranges of ~8 dB have been reported, while the dynamic range on the population level has been estimated to be 10.7 dB, which was comparable to those of monopolar electrical SGN stimulation and bipolar electrical SGN stimulation (Dieter *et al*, 2019). However, two aspects should be considered when comparing the dynamic ranges in this study: First, in most cases of optogenetic stimulation neural responses were not saturated, indicating that the true dynamic range has been underestimated. Second, estimating the dynamic range in response to artificial SGN stimulation is quite tricky: The dynamic range of optogenetic stimulation is based on power, while the dynamic range of electrical stimulation is calculated based on current amplitude, which leads to a difference in the dynamic range calculation by a factor of two. While energy directly relates to membrane depolarization in the case of optogenetic stimulation, the unit relating stimulus intensity to membrane depolarization might be charge rather than amplitude in the case of electrical stimulation. When calculating the electrical dynamic range based on charge (current amplitude multiplied by pulse duration), the dynamic range would be half as large as reported, and thus be surpassed by optogenetic SGN stimulation. Answering the question how many discernible intensity steps can be coded optogenetically will also require behavioral experiments. Increasing irradiance of SGNs by maximizing power output and optimizing positioning of the emitter as well as increased light sensitivity of future ChRs in SGNs will broaden the dynamic range by maxing out optogenetic stimulation. Nonetheless, energy budget as well as potential heating and phototoxicity must be considered. However, experimental data suggest that optogenetic SGN stimulation offers an output dynamic range that is at least as broad as for electrical stimulation.

### Temporal properties of optogenetic SGN activation

Another important objective for optogenetic sound encoding is to enable SGN firing at high rates and with high temporal precision. During strong sound stimulation, SGNs fire at rates in the range of few hundreds of Hz and achieve sub‐millisecond precision of spike timing, which is critical for auditory function (Heil & Peterson, 2015). The temporal properties of optogenetic SGN activation primarily depend on the kinetics of the opsin, mainly limited by the closing kinetics of the ChRs after light‐off. Efforts to speed up ChRs have successfully used mutagenesis of previously identified ChRs such as fast Chrimson variants (Klapoetke *et al*, 2014; Mager *et al*, 2018; Oda *et al*, 2018) as well as identification of naturally occurring ChRs such as Chronos (Gunaydin *et al*, 2010; Klapoetke *et al*, 2014; Mager *et al*, 2018; Oda *et al*, 2018; for a summary, see table 1). The temporal properties of optogenetic SGN activation have often been approximated by recordings of oABRs and single units from the auditory nerve.

**Figure 3:**
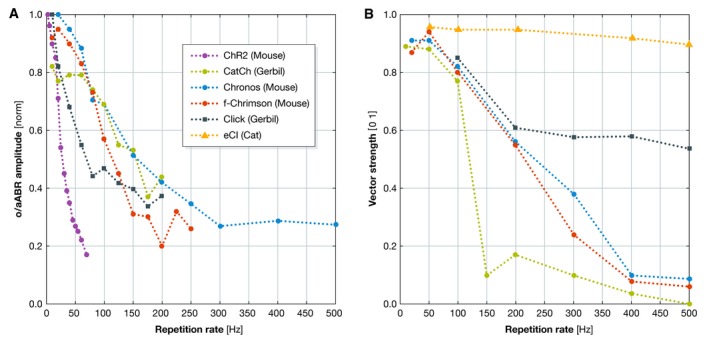
Temporal precision of cochlear optogenetics (A) oABR amplitude as a function of stimulation rate. Since the signal amplitude scales with the amount and synchronization of recruited neurons, it can serve as an estimate for temporal precision. (B) Mean vector strength as a function of stimulation rate. Vector strength quantifies temporal precision of action potentials in individual neurons. Both the population level (A) and the level of single SGNs (B) demonstrate that temporal precision depends on the opsin mediating auditory nerve activation. Figure summarizes data published by Hartmann and Klinke (1990), Hernandez *et al* (2014), Keppeler *et al* (2018), Mager *et al* (2018), and Wrobel *et al* (2018).Source data are available online for this figure.

Upon optical SGN stimulation with light pulses of increasing frequency, wave I of oABRs—which originates from auditory nerve activation (Henry, 1979)—showed a decrease in amplitude and an increase in latency. Since the ABR originates from synchronized firing of individual neurons, both the number of recruited neurons and the synchronization of their firing to the stimulus contribute to the ABR amplitude. Thus, the decreased amplitude likely reflects less reliable SGN activation at higher stimulation rates, both in terms of spike timing and in terms of recruited SGNs (Hernandez *et al*, 2014; Keppeler *et al*, 2018; Mager *et al*, 2018; Wrobel *et al*, 2018). While oABRs vanished at stimulation rates beyond 70 Hz in ChR2‐transgenic animals (Hernandez *et al*, 2014), CatCh (Wrobel *et al*, 2018) and f‐Chrimson (Mager *et al*, 2018) facilitated sizable oABRs even at rates up to 200 Hz (Fig 3A). In mice injected with trafficking‐optimized Chronos‐ES/TS, responses could even be detected up to stimulation rates of 1,000 Hz, although the brevity of the averaged potential at these rates precludes assessment of propagated activity, since only oABR wave I could reliably be analyzed in the response window of 1 ms (Keppeler *et al*, 2018).

To read out spike timing directly, *in vivo* recordings from individual SGNs have been performed upon optogenetic stimulation. Here, the action potentials of single auditory nerve fibers have been recorded with sharp electrodes while stimulating the auditory nerve optogenetically via an optical fiber placed in the round window. Temporal fidelity has been assessed by the jitter of the first spike upon high intensity optical SGN stimulation (~20 mW for ChR2/CatCh, ~10 mW for Chrimson, ~30 mW for Chronos). In transgenic mice expressing ChR2, the jitter amounts to 0.28 ms (Hernandez *et al*, 2014), whereas in CatCh expressing gerbils, it was 0.26 or 1.66 ms for SGNs responding with a single or with multiple spikes, respectively (light pulses were presented at a stimulus rate at 10 Hz; Wrobel *et al*, 2018). In f‐Chrimson and Chronos‐ES/TS expressing SGNs, the jitter amounted to 0.26 and ~0.2 ms (measured at 50 and 20 Hz, respectively; SGNs typically responded with only 1 spike; Keppeler *et al*, 2018; Mager *et al*, 2018). This compares to jitter upon acoustic stimulation using pure tones presented at the characteristic frequency of typically 0.5–1 ms (even though individual neurons with a jitter as small as 0.1 ms were found; Heil & Irvine, 1997; Buran *et al*, 2010; Huet *et al*, 2016) and 0.01–0.03 ms for electrical stimulation (van den Honert & Stypulkowski, 1984).

A second measure for temporal precision of coding—the vector strength—describes the quality of phase‐locking of neuronal responses to the stimulus. It was shown that vector strength of *in vivo* recorded SGNs decreases with increasing stimulation rates in an opsin‐dependent manner. In CatCh‐injected gerbils, vector strength remained high up to rates of 100 Hz but sharply decreased thereafter, even though significant vector strength for stimulation rates up to 240 Hz has been observed in some neurons (Wrobel *et al*, 2018). Using f‐Chrimson (Mager *et al*, 2018) or Chronos (Keppeler *et al*, 2018), significant vector strength has been achieved up to a few hundred Hz on average (Fig 3B). Using Chronos, individual SGNs could even follow stimulation rates up to 1 kHz to some extent (Keppeler *et al*, 2018). However, even the vector strength reported for the fastest opsin—Chronos—did not yet reach the temporal precision achieved with stimulation by acoustic clicks (Wrobel *et al*, 2018) or electrical stimulation (Hartmann & Klinke, 1990).

Finally, spike probability of light‐mediated SGN firing dropped sharply beyond 100 Hz in the case of CatCh, whereas f‐Chrimson and Chronos enable firing to every third light pulse at 200 Hz stimulation rate (Keppeler *et al*, 2018; Mager *et al*, 2018; Wrobel *et al*, 2018). However, for a subset of SGNs in CatCh‐injected gerbils, sustained action potential firing at rates comparable to acoustic stimulation has been reported up to stimulation rates of 500 Hz (Wrobel *et al*, 2018).

Taken together, the use of ultrafast opsins such as f‐Chrimson and Chronos for cochlear optogenetics increases the bandwidth of temporal coding tremendously. Ultrafast optogenetic SGN stimulation approaches steady‐state firing rates of the auditory nerve (200–300 Hz), but the temporal precision of natural sound encoding (Liberman, 1978) has not yet been achieved. Future studies should thus involve opsins with improved kinetics that might reach physiological response properties of the auditory nerve. Finally, even if limitations of temporal precision might not be fully overcome, it might be possible that the limited temporal precision might be compensated on the population level, where information is encoded by several SGNs at a time (e.g., Keppeler *et al*, 2018).

### Cochlear optogenetics for hearing restoration

In order to be considered as an alternative method for hearing restoration, optogenetic SGN stimulation must also be perceptually relevant, independent of hair cell function (i.e., functional in the deafened cochlea), and stable over long periods of time.

Perception of optogenetic SGN stimulation has been demonstrated using a paradigm of negative reinforced learning in Mongolian gerbils that have been implanted with fiber‐based single‐channel oCIs: Optogenetically transduced animals learned to robustly indicate perception of optical stimuli via locomotion within typically three training sessions, proving the behavioral relevance of cochlear optogenetics (Wrobel *et al*, 2018). Further, animals could transfer the behavior from optical to acoustic SGN stimulation within the first training session, suggesting generalization between the perception of auditory and optogenetic stimulation (Wrobel *et al*, 2018). Behaviorally relevant perception of optogenetics in the auditory system was also demonstrated in a study conducted at a higher station of the auditory pathway: Upon viral transduction of auditory midbrain neurons with ChR2 or Chronos, mice reported perception of optogenetic midbrain stimulation via locomotion (Guo *et al*, 2015).

Toward hearing restoration, multiple studies have demonstrated the feasibility of optogenetic SGN stimulation in the deafened cochlea. In ChR‐2 transgenic mice, oABRs could still be evoked after auditory function has been abolished due to subcutaneous furosemide injection (Hernandez *et al*, 2014), which collapses the endocochlear potential and thus mechanotransduction by hair cells (Sewell, 1984; Hernandez *et al*, 2014). Furthermore, oABRs have been successfully evoked in a mouse model of human deafness (DFNB9; Hernandez *et al*, 2014), which is characterized by severely impaired transmitter release from inner hair cells and the absence of aABRs (Pangrsic *et al*, 2010). In a different study, oABRs have been evoked in C57BL/6J mice at 9 months of age (Mager *et al*, 2018), which served as a model of age‐related hearing loss (Shnerson *et al*, 1981). Finally, optogenetic SGN stimulation has been shown to re‐activate the deafened auditory system in a gerbil model of sensorineural deafness (aminoglycoside‐induced loss of hair cells) both on a physiological level and on a behavioral level (Wrobel *et al*, 2018).

Another important aspect when considering optogenetics for hearing restoration is the stability of opsin expression over time. Even though studies covering the whole lifespan of animals have not been performed yet, it has been shown that viral transduction facilitated robust oABRs and stable expression of f‐Chrimson in mice at least 9 months after injection, while the density of SGNs was unaltered in the injected as compared to the non‐injected ear (Mager *et al*, 2018). A different study found similar oABR appearance and expression levels of Chronos in the auditory nerve of mice 6–18 weeks after injection (Duarte *et al*, 2018). These findings are supported by regular oABR measurements of gerbils implanted with optical fibers, which showed stable responses to optogenetic SGN stimulation over more than 100 days after implantation (Wrobel *et al*, 2018). Hence, the functionality of cochlear optogenetics in animal models of deafness has been demonstrated over months. Future experiments need to integrate these approaches and demonstrate the spectral specificity of cochlear optogenetics on a behavioral level, ideally in a longitudinal way and combined with biosafety studies.

## Multi‐channel optoelectronic stimulators—a prerequisite for cochlear optogenetics

In parallel to the biomedical advancements of cochlear optogenetics, also the technologically demanding engineering of multi‐channel oCIs is rapidly progressing. The design requirements include power‐efficient optical emitters with narrow beam profile, integrated into a flexible carrier at large numbers (tens to hundreds; Jeschke & Moser, 2015; Moser, 2015). Furthermore, implants should fit the limited intracochlear space, be stiff enough to allow for implantation but sufficiently flexible to follow the cochlea's curvature, and to avoid cochlear trauma. Encapsulation of the implants should be electrically insulating, transparent, biocompatible, and provide long‐term stability, as CIs need to work over decades.

### LED‐based (active) oCIs

**Figure 4:**
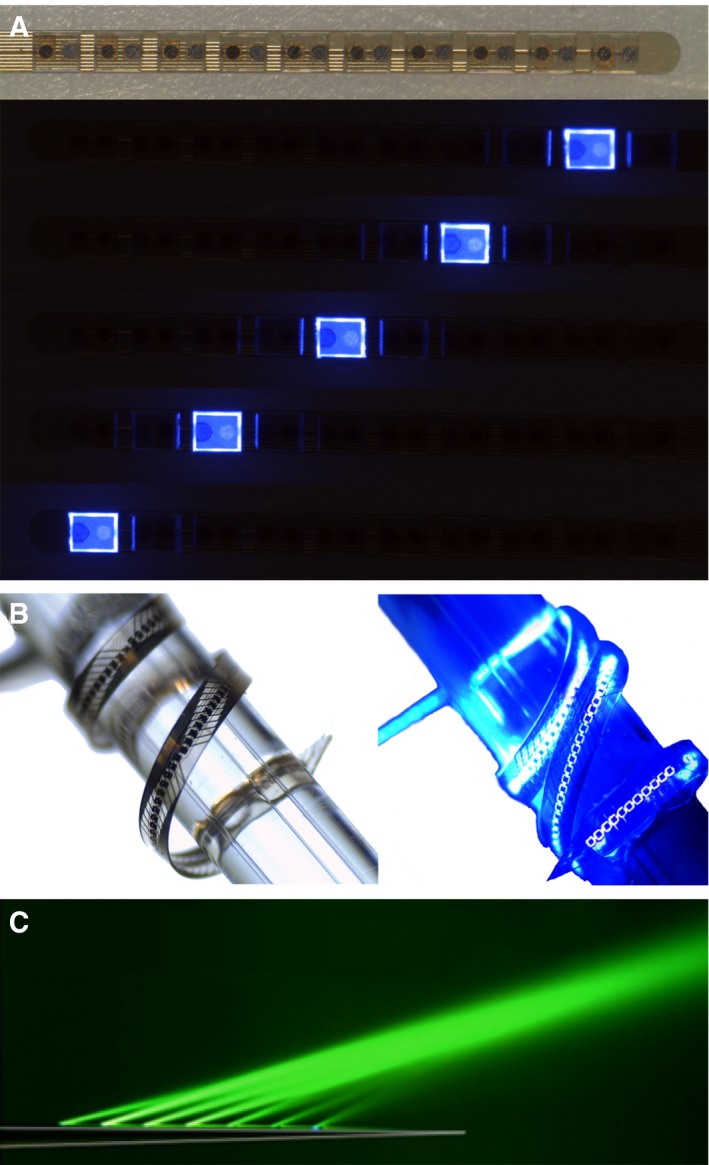
Multi‐channel optogenetic stimulators (A) oCI housing 10 commercially available LEDs. (B) oCI housing 144 thin‐film μLEDs. (C) Gold‐coated, multi‐point emitting optical fiber with seven outcoupling windows. Images are taken from Klein *et al* (2018), Pisanello *et al* (2014), and Schwaerzle *et al* (2016).Source data are available online for this figure.

One approach of oCI fabrication used commercially available light‐emitting diodes (LEDs; fabricated by Cree Europe GmbH; emission peak: 460 nm), which were integrated on a flexible polyimide substrate of 20 mm length and 0.24 mm width, allowing for a bending radius of 1 mm (Fig 4A; Schwaerzle *et al*, 2016). Ten LEDs of 220 × 270 μm with a pitch of either 350 or 500 μm were employed and could be individually addressed. Driven with a current of 5 mA, the optical power of LEDs amounted to ~0.3 mW (and could be as high as 1.9 mW when driven with 45 mA at a duty cycle of 10%), which—normalized to the surface of the LED—amounts to a power density of ~32 mW/mm² (i.e. 320 mW/mm^2^ during the pulses; and thus suffices to drive most ChRs in close proximity; Deisseroth & Hegemann, 2017), while the temperature increase in the oCI probe amounted to 1.67°C (driven at a duty cycle of 10% while placed on agarose gel; Schwaerzle *et al*, 2016). In a different study, an oCI housing 15 LEDs (1 × 0.6 mm; max. 34 mW at 470 nm) embedded in biocompatible silicone has been realized and implanted into a human scala tympani model with insertion forces comparable to commercially available eCIs (Xu *et al*, 2018). Besides application for cochlear optogenetics, wireless controllable LEDs with ultraviolet (100 × 100 μm), blue, green, yellow, and red (220 × 270 μm each) emission peaks have been developed for optogenetic stimulation of the central nervous system *in vivo* (Shin *et al*, 2017)*,* which might be utilized when developing oCIs for opsins with shifted peak action spectra in the future.

To further decrease the size of light emitters, and thus increase the number of emitters that can be placed on an oCI, custom‐made thin‐film μLEDs with dimensions as small as 50 × 50 μm have been developed based on gallium nitride (GaN; Goßler *et al*, 2014; Klein *et al*, 2018). GaN, a biocompatible semiconductor with high chemical stability (lifetimes of 11 years and beyond), is commonly used to manufacture LEDs emitting light from the green to ultraviolet spectrum with high brightness with power efficiencies as high as 60% (Laubsch *et al*, 2010; Jewett *et al*, 2012; Alt *et al*, 2017). Parallelized fabrication of the first two designs of optical cochlear implants housing GaN‐based μLEDs for rodent applications at the level of wafers—i.e., substrates allowing for μLED manufacturing on a larger scale as individual μLEDs—has been described in 2014 (Goßler *et al*, 2014). Such an oCI with a total width of 230 μm and a length of 5 mm housed four μLEDs of 50 × 50 × 15 μm and could be successfully implanted into a mouse cochlea via the round window. The output power of these four μLEDs, driven at a current of 1 mA, amounted to 60 μW (at a wavelength of 405 nm; Goßler *et al*, 2014). Another active oCI implementation had a width of 380 μm and a length of 5 mm. It housed 15 μLEDs of 150 × 150 × 15 μm of which up to three could be individually addressed and has successfully be inserted into a model of a rat cochlea (Goßler *et al*, 2014). Based on the established wafer‐level processes, oCIs of 350 μm width and 15 mm length with a total of 144 individually addressable μLEDs of 60 × 60 μm have been engineered recently (Fig 4B; Klein *et al*, 2018). In those new oCIs, the carrier substrate for the light emitters is fully epoxy‐based, which minimizes thermomechanical bending and renders the implants translucent and biocompatible. Besides the enormous upscale of individual light emitters, also the optical power has been substantially increased: When driving individual μLEDs at 10 mA, the output power (at a wavelength of 462 nm) amounted to 0.82 mW, which corresponds to a power density of 407 mW/mm². Finally, a maximum temperature increase of 1°C was measured when driving the μLEDs with DC pulses of ~20 ms of duration and 10 mA intensity when the implant was placed on agarose, which makes these implants suitable for *in vivo* application (Klein *et al*, 2018), considering maximum pulse durations of 1 ms in these applications (maximum stimulation rates will then also depend on the time of μLEDs to cool down to baseline again). The optical properties of these implants have further been enhanced by adding conical concentrators and spherical micro‐lenses onto the emission side of μLEDs (Klein *et al*, 2019). Concentrators and micro‐lenses were fabricated from polydimethylsiloxane, a material which is highly transparent, flexible, and biocompatible, and increased light extraction by 83% and peak intensity by 95% (measured in water; Klein *et al*, 2019). In addition, a modest improvement in the beam profile was achieved. The increase in light extraction provides higher light intensities and thus evokes more robust activation of ChRs, but also minimizes power consumption and thus temperature increase in the target tissue when using identical illumination intensities as without micro‐lenses.

### Waveguide‐based (passive) oCIs

In contrast to active optoelectronic light emitters near the neural target tissue, passive optogenetic stimulators deliver light originating from external sources, such as LEDs or laser diodes, by using waveguides. Passive stimulators have the advantage to spatially separate optoelectronics from the cochlea, which enables hermetically sealed encapsulation and lowers the requirements regarding biocompatibility, heat generation, and size of the light emitter. However, they face light loss at the emitter–waveguide interface as well as along the waveguides (Alt *et al*, 2017). A first passive oCI composed of eight glass fibers of 20/25 μm diameter (core/cladding) embedded in silicone elastomer has been realized. Insertion forces for this device measured in a 2D cochlea model were comparable to conventional CI insertion forces, and atraumatic implantation up to 20 mm depth was possible in human temporal bones (Balster *et al*, 2014). An increase in the number of stimulation channels of waveguide‐based implants might be achieved by using single‐mode fibers, which are available with core diameters of 2–10 μm, rather than the multi‐mode fibers used in this study, which typically have diameters in the range of tens to hundreds of micrometers (Balster *et al*, 2014; Alt *et al*, 2017). Single‐mode fibers have the further advantage of lower numerical apertures, which enable higher spatial confinement of the emitted light at the target tissue. However, the coupling of light from an emitter into the fiber is more challenging than with multi‐mode fibers (Alt *et al*, 2017). Furthermore, polymer‐based waveguides which have been manufactured with core thicknesses below 10 μm offer an alternative approach, especially when considering mechanical properties, i.e., flexibility to wind along the cochlear spiral, and the number of independent stimulation channels, for which the waveguide dimensions are of critical importance (Zorzos *et al*, 2010; Alt *et al*, 2017; Xu *et al*, 2018). However, even state‐of‐the‐art polymer waveguides do not reach the outstanding light propagation of glass fibers. With a different approach, locally precise optical stimulation of the central nervous system has been realized by integrating up to seven optical windows into a gold‐coated (and thus internally reflective) optical fiber of 125 μm diameter. Different optical windows could then be selected for outcoupling of the light by varying the angle at which light is coupled into the fiber (Fig 4C; Pisanello *et al*, 2014).

Taken together, the development of spatially selective multi‐channel optical stimulation of neural tissue is an active area of research and there are various possible strategies that qualify as candidates for oCIs. Even though functionality and stability of multi‐channel oCIs *in vivo* remain to be demonstrated, state‐of‐the‐art optogenetic stimulators meet the basic requirements of future oCIs regarding size, number of emitters, and light output while considering thermomechanical properties such as flexibility and heating of the implants.

## Future objectives for clinical translation of cochlear optogenetics and challenges ahead

### Optimizing optogenetic manipulation of SGNs

Optogenetic manipulation of the auditory nerve requires robust opsin expression in the majority of SGNs across all cochlear turns, as demonstrated by the positive correlation of oABR amplitudes with the fraction of opsin expressing SGNs in various model systems (Hernandez *et al*, 2014; Duarte *et al*, 2018; Wrobel *et al*, 2018). Even though several methods have been developed for SGN transduction, all of them have their drawbacks regarding clinical translation.

Transuterine virus injections of AAV2/6 carrying plasmids encoding the opsin CatCh in mice transduced 40–60% of SGNs in the basal turn of the cochlea, while expression in SGNs of the middle and apical turn was below 10% (Hernandez *et al*, 2014). Although this approach was an important step toward viral opsin delivery to auditory neurons and has several advantages over transgenic animal models, both the delivery method and the heterogeneous opsin expression invalidate this technique for applications in a clinical setting. Early postnatal injections into the mouse cochlea achieve homogeneous opsin expression along the cochlear axis in 60–90% of SGNs (Duarte *et al*, 2018; Keppeler *et al*, 2018; Mager *et al*, 2018). Importantly, SGN density in the injected ears was unaltered as compared to non‐injected ears, indicating largely atraumatic transduction, but opsin expression was also reported for the contralateral, non‐injected ears (Keppeler *et al*, 2018; Mager *et al*, 2018), probably due to viral spread via the cochlear or endolymphatic ducts, or via the temporal bone marrow space (Kho *et al*, 2000). Furthermore, injections were performed before hearing onset into immature cochleae, which are weakly ossified (Kraus & Aulbach‐Kraus, 1981). A different method will probably be needed for virus delivery to the spiral ganglion in humans, which is housed in a cochlea that is almost fully developed and highly ossified at birth (Haith, 1986). In a more translational approach, intramodiolar virus injections have directly been targeted to the auditory nerve of adult Mongolian gerbils (Wrobel *et al*, 2018). While this method achieved homogeneous opsin expression across all cochlear turns and was restricted to the injected cochlea, it suffers from different drawbacks: Transduction efficiency was relatively low when compared to early postnatal injections. Only about half of the injected animals showed oABRs, and in these animals, the transduction rate amounted to only ~30%. Furthermore, a reduction in SGN density was observed across all cochlear turns (~25%), which might be caused by the pressure injection into the restricted volume of Rosenthal's canal (Wrobel *et al*, 2018). Thus, future preclinical studies should focus on the development of atraumatic and reliable methods for virus delivery to auditory neurons in the mature cochlea.

Next, safe and efficient viral vectors are required. AAVs appear to be highly promising candidates, since they have little potential for virus‐related harm in the transduced tissue, while being characterized by an intrinsically high neural tropism, long‐term availability of the desired transgene, and high expression levels (Willett & Bennett, 2013; Ahmed *et al*, 2017; Hudry & Vandenberghe, 2019; Lotfinia *et al*, 2019). Experimentally, AAVs have been used in several studies to genetically restore auditory function, and even reached clinical trials for the treatment of various other disorders, including retinal dysfunction (Akil *et al*, 2012; Askew *et al*, 2015; Landegger *et al*, 2017; Pan *et al*, 2017; Suzuki *et al*, 2017; Al‐Moyed, 2019; Hudry & Vandenberghe, 2019; Lotfinia *et al*, 2019). In fact, a first AAV‐mediated gene therapy for vision restoration (Luxturna) has recently been FDA‐approved (Keeler & Flotte, 2019; Lotfinia *et al*, 2019). Furthermore, AAV optimization by *in silico* reconstruction and targeted evolution resulted in powerful AAV variants such as Anc80, AAV2/7m8, PHP.B, and PHP.eB, which are characterized by increased efficiency of viral transduction in various tissues, including cochlear hair cells and SGNs (Dalkara *et al*, 2013; Zinn *et al*, 2015; Deverman *et al*, 2016; Chan *et al*, 2017; Landegger *et al*, 2017; Keppeler *et al*, 2018). Careful evaluation of each viral vector in several preclinical (animal) models is needed: Highly potent AAV variants can lead to harmful effects when systemically applied in high doses (Hordeaux *et al*, 2018). While the transduction of target neurons should be highly effective, it should also be SGN‐specific, and transducing off‐target cells must be avoided. Local, intramodiolar virus administration in adult gerbils led to specific opsin expression in SGNs of the injected ear only, but a rigorous screening of various tissues across the body has not yet been performed (Wrobel *et al*, 2018). Besides the administration route and the viral tropism, also the choice of cell type‐specific promoters governs specificity of viral transduction. Molecular SGN profiling will facilitate the identification of suitable SGN‐specific promoters (Shrestha *et al*, 2018; Sun *et al*, 2018).

Once the transgene is delivered to its target, the next concern is stable and selective expression in the plasma membrane. Trafficking of microbial opsins to the plasma membrane can be limiting (Keppeler *et al*, 2018; Wrobel *et al*, 2018), and protein accumulation in the endoplasmic reticulum can lead to proteostatic stress. Toward this end, several approaches have demonstrated improvement in the past years: First, an export signal derived from inward rectifying potassium channels (*K*_ir_) was shown to enhance export of the synthetized protein from the endoplasmic reticulum and increase the number of proteins integrated into the cell membrane (Ma *et al*, 2001; Gradinaru *et al*, 2010). Second, a trafficking signal, also derived from the K_ir_ family, was shown to increase membrane localization of the synthetized protein (Stockklausner *et al*, 2001; Gradinaru *et al*, 2010). The combination of these export and trafficking signals was recently shown to improve the targeting of opsins to the cell membrane of HEK cells, primary hippocampal cell cultures, and SGNs (Keppeler *et al*, 2018). Furthermore, the implementation of these signals when transducing the auditory nerve optogenetically increased the success rate of SGN expression from ~50% to ~95% and halved the light thresholds of oABRs (Keppeler *et al*, 2018). While expression levels could further be boosted by various enhancing elements, careful titration is needed to avoid overexpression and resulting detrimental effects such as cytotoxicity (Powell *et al*, 2015).

Finally, the question remains which opsin should be employed for most natural sound encoding in SGNs. Ideally, the opsin of choice should combine fast kinetics (to enable physiological firing rates), large ion conductance (to allow for robust photocurrents and minimize needed protein in the cell membrane), red‐shifted action spectrum (to minimize phototoxicity), and confer high light sensitivity to SGNs (to reduce radiation and energy requirements). Temporal fidelity of high‐frequency spiking is mainly limited by the closing kinetics of the channel, and opsins with the fastest closing kinetics known to date include the light‐activated ChR Chronos (τ_off_ = 3.6 ms at room temperature and < 1 ms at physiological temperature) and the ChR2 mutant CheTa (4.4 ms at room temperature) as well as the red light‐activated Chrimson variants f‐Chrimson (5.7 ms at room temperature and 3.2 ms at physiological temperature) and vf‐Chrimson (2.7 ms at room temperature and 1.6 ms at physiological temperature), which have been sped up by directed mutations in helix 6 (Gunaydin *et al*, 2010; Klapoetke *et al*, 2014; Keppeler *et al*, 2018; Mager *et al*, 2018). These helix 6 mutations could also be implemented in other opsins, such as Chronos, to further improve channel kinetics. Fast closing kinetics comes at the price of reduced neural light sensitivity, as for the same level of expression, the shorter lifetime of the open channel leads to smaller photocurrents (Mager *et al*, 2018). Thus, future studies will need to balance between channel kinetics and light sensitivity, in order to enable optogenetic control of SGNs at natural firing rates and at reasonable light thresholds. Finally, the ideal opsin for cochlear optogenetics should have an action spectrum that is shifted toward larger wavelengths in the range of red light for several reasons: First, red light is reported to be less phototoxic than blue light and would therefore be preferred for safety reasons (Kerstein *et al*, 2014; Mager *et al*, 2018). Second, red light is less scattered and absorbed and thus penetrates deeper into biological tissue, lowering the illumination intensities needed for opsin activation and potentially enabling better spatial confinement of optical SGN stimulation (Jacques, 2013).

### Gene therapy

Adeno‐associated viruses have been proven to be safe in most studies, enabling genetic modification of target cells without harming tissue or causing pathologies. AAV‐mediated gene therapy to restore visual function in patients suffering from Leber's congenital amaurosis, which has recently received FDA approval, serves as an important model for sensory gene therapy. Importantly, aside from a transient inflammatory response, no adverse effects were reported, restoration has lasted for several years, and AAV administration to the second eye has proven successful, indicating stability and reliability of the treatment despite potential neutralizing antibodies upon AAV exposure (Simonelli *et al*, 2010; Bennett *et al*, 2012). Nonetheless, other clinical gene therapy studies, mostly using systemic AAV application, have reported problems, highlighting the importance to perform biosafety studies for each individual virus, transgene, titer, and administration route (Hinderer *et al*, 2018; Hordeaux *et al*, 2018; Rabinowitz *et al*, 2019). Most obviously, possible immune responses against the viral vector or the transgene need to be evaluated and combated if present. In general, the cochlea, much like the retina, is considered to be immune‐privileged, primarily owing to the blood–labyrinth barrier, and hence, the hope is that local administration can avoid immune responses. A further aspect requiring consideration is the potential presence of neutralizing antibodies to AAVs that can hinder or preclude successful AAV‐based gene therapy (Mendoza *et al*, 2017; Hudry & Vandenberghe, 2019). Such humoral immunity to the vector can result from natural AAV exposure before the gene therapy and needs to be identified to evaluate candidacy for the gene therapy. Neutralizing antibodies to AAVs can also arise after the first virus administration, then challenging a second injection, e.g., in the other ear for rehabilitation by bilateral oCI (Mendoza *et al*, 2017; Hudry & Vandenberghe, 2019). Strategies to overcome this limitation include the use of empty capsids that bind neutralizing antibodies, the use of different serotypes, engineered capsids with reduced sensitivity to neutralizing antibodies, and shielding of the viral capsids (Mingozzi & High, 2013; Lotfinia *et al*, 2019).

Next to biosafety, the oCI approach relies on long‐term stability of the transgene at no harm to the target structure. Since optogenetics is a quite recent technique, longitudinal data of long‐term opsin expression are only available from a few studies: In the mouse cochlea, the expression of f‐Chrimson in SNGs did not cause any cell loss and was stable over at least 9 months (Mager *et al*, 2018). Auditory neurons in the brainstem of rats showed robust opsin expression in the absence of cytotoxicity even 12–18 months after virus injection (Shimano *et al*, 2013). In the visual systems, where optogenetic restoration of function is more advanced than in the field of inner ear therapy, opsins neither had toxic effects in the mouse retina up to 12 months after injection, nor in human retinal organoids (Bi *et al*, 2006; Busskamp *et al*, 2010; Doroudchi *et al*, 2011; Garita‐Hernandez *et al*, 2018). In fact, two clinical trials have recently been approved by the FDA for optogenetics‐based vision restoration (NCT02556736 and NCT03326336), highlighting the potential of optogenetics for sensory restoration (Garita‐Hernandez *et al*, 2018). However, a few studies have reported opsin aggregates, which might result from overexpression, affirming the importance that each construct must be optimized regarding parameters such as gene dosage, promoter, and trafficking signals, in order to achieve appropriate expression levels for its corresponding application, avoid toxicity, and ensure a high level of safety for the patient (Gradinaru *et al*, 2008; Diester *et al*, 2011; Allen *et al*, 2015).

### Energy requirements for optogenetic SGN activation

In order to enable safe and energy‐efficient optical stimulation of auditory neurons, reasonable thresholds for optogenetic SGN activation must be achieved. This is of uppermost importance when considering tissue heating and phototoxicity upon chronic illumination of the cochlea, and also inevitable to achieve reasonable battery lifetimes of future optical CIs, which should last at least 1 day. Thus, the radiant energy required for optogenetic SGN activation should be minimized for reasons of biosafety and the energy per pulse should ideally be comparable to energy requirements in eCIs (~0.2 μJ for a pulse of 80 μs) in order for the oCI to arrive at similar battery lifetimes (Zierhofer *et al*, 1995; Hernandez *et al*, 2014). Activation thresholds of cochlear optogenetics mainly depend on the opsins and their expression level: Energy thresholds for optogenetic SGN activation have been determined for ChR2 (2.2 μJ, oABRs; Hernandez *et al*, 2014), Chronos (6–9 μJ, oABRs, ICC multi‐units; Duarte *et al*, 2018; Keppeler *et al*, 2018), Chronos‐ES/TS (4.6 μJ, oABRs; Keppeler *et al*, 2018), f‐Chrimson (0.5–1 μJ, oABRs; Mager *et al*, 2018), and CatCh (1.8–4.6 μJ, oABRs, cortical single units, ICC multi‐units, and behavioral analysis; Wrobel *et al*, 2018; Dieter *et al*, 2019). While these values still exceed the ones of eCIs, more appropriate intracochlear positioning of emitters toward the spiral ganglion is likely to lower the energy requirements, as indicated by modeling studies (Wrobel *et al*, 2018; Dieter *et al*, 2019). Since the field of optogenetics is developing quite rapidly, and optogenetic tools with optimized characteristics are frequently reported, ChRs conferring even higher light sensitivity to neurons might become available for future studies. Greater transduction rates of SGNs due to optimized viruses or more efficient promoters as well as improved membrane trafficking of ChRs could also contribute to increased light sensitivity of the auditory nerve (Keppeler *et al*, 2018). Finally, the required energy for optical sound encoding will be largely governed by the employed coding strategy, which needs to balance the duration of individual light pulses (to evoke robust responses) and their intensity over time (to encode for loudness), and assemble different pulses with varying repetition rates (to encode temporal information) delivered via multiple stimulation channels (to encode spectral information) in order to meaningfully mimic acoustic signals.

### Medical device

Besides the biosafety of molecular tools, also long‐term safety and stability of optical SGN stimulation require critical assessment. Light pulses with repetition rates of few hundreds of Hz, which illuminate the cochlea over decades (at least during the time in which the user is awake) and might lead to phototoxicity (for blue light), heating, or changes in neural properties such as long‐term potentiation, need to be evaluated (Zhang & Oertner, 2007; Delbeke *et al*, 2017; Senova *et al*, 2017). While irradiances of up to ~75 mW/mm² are considered to be safe for optogenetic applications *in vivo*, some studies pushed the limit even further: While tissue heating has been reported for stimulation intensities of 200 mW/mm² (blue light: 0.1°C, red light: 0.3°C), phototoxic effects such as cell loss or apoptosis were absent at irradiances up to 600 mW/mm² (Cardin *et al*, 2010; Senova *et al*, 2017). These studies have been done with relatively long illumination (~5 ms of pulse duration), while light pulses used for SGN excitation typically are around ~1 ms and might even be shortened with improved opsins. Independent of these values and absolute safety thresholds—which still need to be defined for cochlear optogenetics—it is important to keep in mind that safety limits heavily depend on the wavelength needed to excite the opsin of choice, since blue light has a higher potential of phototoxicity: In fact, safety limits for retina exposure to orange light are three orders of magnitude higher as compared to blue light, thus enabling optogenetic stimulation at higher intensities when using red‐shifted ChR2s (European Commission, 2006; International Commission on Non‐Ionizing Radiation Protection, 2013a,b; Duebel *et al*, 2015; Sengupta *et al*, 2016).

In terms of long‐term stability, the eCI is a tough benchmark to meet, given the robust materials and encapsulation technology used: With a stimulator hermetically sealed in titanium housing, noble metal electrode contacts, and wires embedded in silicone, the eCI is typically stable for decades. The life expectancy of semiconductor emitters is substantial. For example, claims range from 50,000 to 100,000 h (Laubsch *et al*, 2010), which, dependent on the duty cycle, is promising for long‐term SGN stimulation. For the “active” oCI, the encapsulation of the implanted array of optoelectronic emitters will be of critical importance, as water vapor penetration can lead to damage.

### Sound coding strategies for sound encoding by oCIs

Optical sound encoding will take advantage of an increased number of independent stimulation channels consequently scaling up energy consumption. However, the stimulation rates at each channel might be drastically reduced to elicit SGN firing at near‐physiological rates. This seems justified, as the synchronization of SGN firing is less pronounced with optogenetic (Keppeler *et al*, 2018; Mager *et al*, 2018; Wrobel *et al*, 2018) than with electrical (van den Honert & Stypulkowski, 1984; Miller *et al*, 2006) stimulation. Therefore, stochastic activity in single auditory nerve fibers can likely be achieved by optogenetic stimulation at 200–300 Hz without evoking additional desynchronization by driving refractoriness in SGNs with kHz stimulation, as done with current eCIs. Moreover, work by Shannon and others has shown that the gain in eCI performance with high stimulation rates is modest (Shannon *et al*, 2011); hence, the improved spectral coding oCIs might not benefit from higher rates. The coding strategy will need to consider the properties of optogenetic SGN stimulation and the design of the oCI. Pulse durations between 100 and 1,000 μs seem appropriate given that oABR amplitudes grow with pulse duration up to approximately 600–1,000 μs and tend to become smaller for longer pulses, possibly due to accumulating channel inactivation and/or increasing depolarization block of SGNs upon prolonged photo‐depolarization (Keppeler *et al*, 2018; Mager *et al*, 2018; Wrobel *et al*, 2018).

Future optogenetic hearing restoration encompasses the combination of (opto)gene therapy and oCI as a medical device. Sound processors and coding strategies driving multi‐channel optical stimulation need to be tested and benchmarked against multi‐channel eCIs.

Taken together, the future development of the oCI should include longitudinal studies on unwanted side effects such as unspecific opsin expression, immune reactions, opsin‐related cytotoxicity, and phototoxicity in order to guarantee a maximum of safety when translating cochlear optogenetics to humans. These *in vivo* studies could be complemented in post‐mortem experiments involving human tissue, or even in human organoids, as demonstrated for optogenetics stimulation of the retina (Sengupta *et al*, 2016; Koehler *et al*, 2017; Landegger *et al*, 2017; Garita‐Hernandez *et al*, 2018). Even though a long way remains to be gone before oCI technology might enter the clinics, the experimental results and technological developments during the past years raise hopes that optogenetics‐based hearing restoration might overcome the major bottleneck of electrical hearing restoration and thus improve the quality of artificial sound encoding in the future.

**Pending issues**

(i) Integrate multi‐channel oCIs with electrophysiological and behavioral studies *in vivo* in order to cross‐validate the technological feasibility of oCIs and their advantage over the eCI.
(ii) Thorough evaluation of biosafety, including stability and specificity of optogenetic transduction, immune responses, phototoxicity, and lifetime of the implants.
(iii) Optimization of optogenetic tools and their delivery as well as oCI layout for application in humans.
(iv) Development of an adequate processor and coding strategy to drive oCIs.

## Conflict of interest

D.K. and T.M. are co‐founders of the OptoGenTech company.

## Supporting information

Source Data for Figure 3

Source Data for Figure 4
