# Supplementary figures and images for: Towards the optical cochlear implant: optogenetic approaches for hearing restoration
Source: EMBO Mol Med. 2020 Mar 30;12(4):e11618. doi: 10.15252/emmm.201911618 (PMC7136966; doi:10.15252/emmm.201911618)

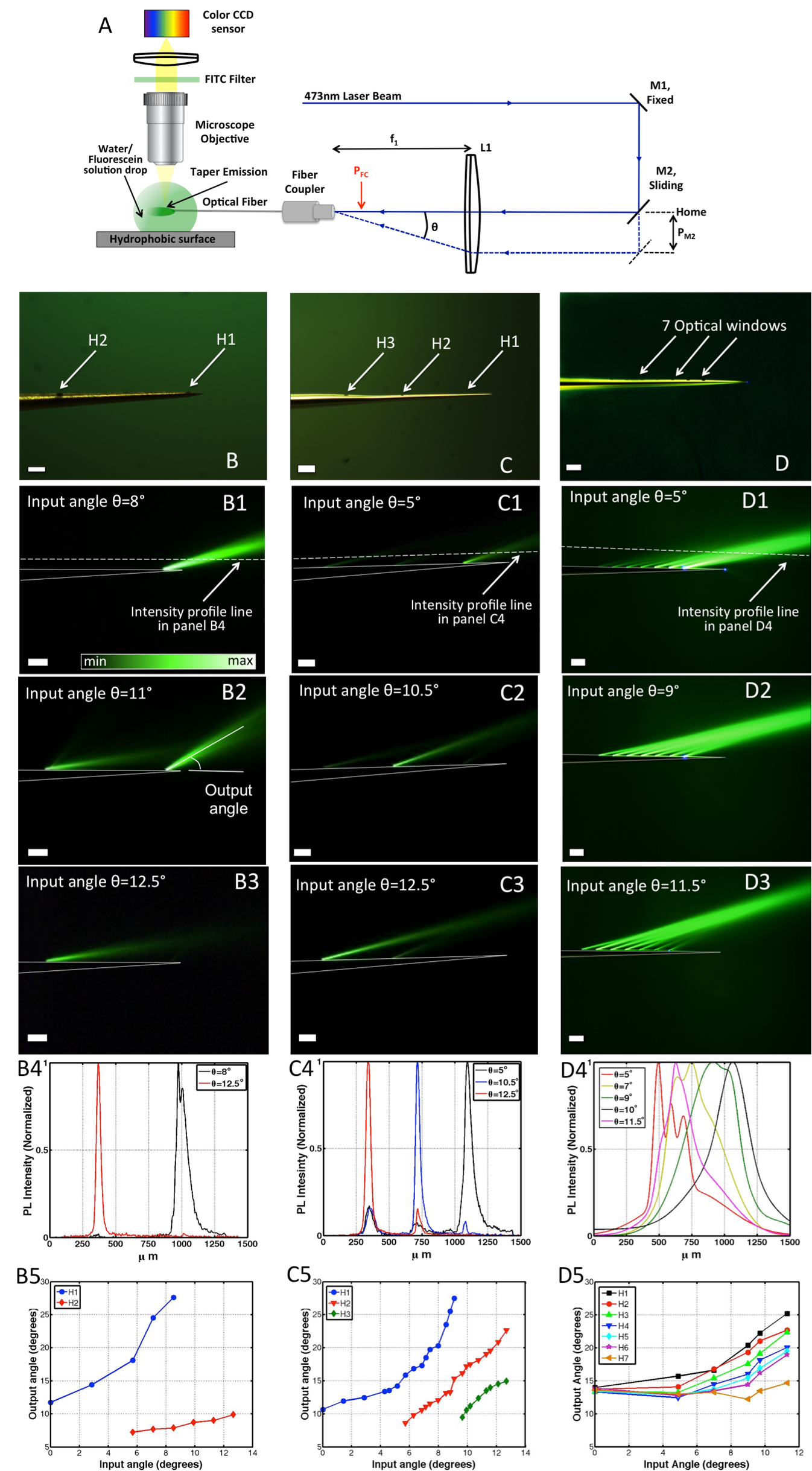

Supplement: Supplementary file 1 — Source Data for Figure 3 [file EMMM-12-e11618-s001.tif]

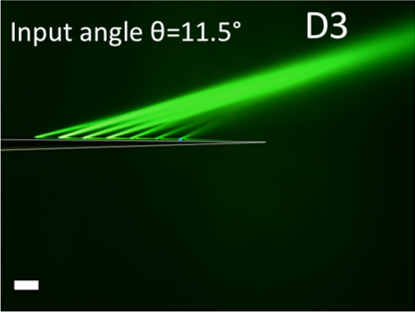

Supplement: Supplementary file 2 — Source Data for Figure 4 [file EMMM-12-e11618-s002.zip › fig4_panelC_1578940239_3.tiff]
